# Proteomic Analysis Reveals a Predominant NFE2L2 (NRF2) Signature in Canonical Pathway and Upstream Regulator Analysis of *Leishmania*-Infected Macrophages

**DOI:** 10.3389/fimmu.2019.01362

**Published:** 2019-06-28

**Authors:** Juliana Perrone Bezerra de Menezes, Ricardo Khouri, Camila Victoria Sousa Oliveira, Antonio Luis de Oliveira Almeida Petersen, Tais Fontoura de Almeida, Flávia R. L. Mendes, Amanda do Amor Divino Rebouças, Amanda Lopes Lorentz, Nívea Farias Luz, Jonilson Berlink Lima, Pablo Ivan Pereira Ramos, Rodrigo Pedro Soares, Jeronimo Nunes Rugani, Gregory A. Buck, Marco Aurélio Krieger, Fabrício Klerynton Marchini, Áislan de Carvalho Vivarini, Ulisses Gazos Lopes, Valéria de Matos Borges, Patricia Sampaio Tavares Veras

**Affiliations:** ^1^Laboratory of Host–Parasite Interaction and Epidemiology, Gonçalo Moniz Institute, Fiocruz–Bahia, Salvador, Brazil; ^2^Laboratory of Vector Born Infectious Diseases, Gonçalo Moniz Institute, Salvador, Brazil; ^3^Department of Pathology and Legal Medicine, Faculty of Medicine, Federal University of Bahia, Salvador, Brazil; ^4^Laboratory of Physiopathology, Federal University of Rio de Janeiro, Macaé, Brazil; ^5^Laboratory of Inflammation and Biomarkers, Gonçalo Moniz Institute, Salvador, Brazil; ^6^Centro de Ciências Biológicas e da Saúde, Federal University of the Western of Bahia, Barreiras, Brazil; ^7^Center for Data and Knowledge Integration for Health, Gonçalo Moniz Institute, Fiocruz-Bahia, Salvador, Brazil; ^8^René Rachou Institute, Fiocruz-Minas Gerais, Belo Horizonte, Brazil; ^9^Department of Microbiology and Immunology, Virginia Commonwealth University, Richmond, VA, United States; ^10^Carlos Chagas Institute, Fiocruz-Paraná, Paraná, Brazil; ^11^Laboratory of Molecular Parasitology, Center of Health Science, Carlos Chagas Filho Biophysics Institute, Federal University of Rio de Janeiro, Rio de Janeiro, Brazil; ^12^National Institute of Science and Technology of Tropical Disease, Patos, Brazil

**Keywords:** *Leishmania*, macrophage, NRF2, CBA mice, iron metabolism

## Abstract

CBA mice macrophages (MØ) control infection by *Leishmania major* and are susceptive to *Leishmania amazonensis*, suggesting that both parasite species induce distinct responses that play important roles in infection outcome. To evaluate the MØ responses to infection arising from these two *Leishmania* species, a proteomic study using a Multidimensional Protein Identification Technology (MudPIT) approach with liquid chromatography tandem mass spectrometry (LC-MS/MS) was carried out on CBA mice bone-marrow MØ (BMMØ). Following SEQUEST analysis, which revealed 2,838 proteins detected in BMMØ, data mining approach found six proteins significantly associated with the tested conditions. To investigate their biological significance, enrichment analysis was performed using Ingenuity Pathway Analysis (IPA). A three steps IPA approach revealed 4 Canonical Pathways (CP) and 7 Upstream Transcriptional Factors (UTFs) strongly associated with the infection process. NRF2 signatures were present in both CPs and UTFs pathways. Proteins involved in iron metabolism, such as heme oxigenase 1 (HO-1) and ferritin besides sequestosome (SQSMT1 or p62) were found in the NRF2 CPs and the NRF2 UTFs. Differences in the involvement of iron metabolism pathway in *Leishmania* infection was revealed by the presence of HO-1 and ferritin. Noteworty, HO-1 was strongly associated with *L. amazonensis* infection, while ferritin was regulated by both species. As expected, higher HO-1 and p62 expressions were validated in *L. amazonensis*-infected BMMØ, in addition to decreased expression of ferritin and nitric oxide production. Moreover, BMMØ incubated with *L. amazonensis* LPG also expressed higher levels of HO-1 in comparison to those stimulated with *L. major* LPG. In addition, *L. amazonensis*-induced uptake of holoTf was higher than that induced by *L. major* in BMMØ, and holoTf was also detected at higher levels in vacuoles induced by *L. amazonensis*. Taken together, these findings indicate that NRF2 pathway activation and increased HO-1 production, together with higher levels of holoTf uptake, may promote permissiveness to *L. amazonensi*s infection. In this context, differences in protein signatures triggered in the host by *L. amazonensis* and *L. major* infection could drive the outcomes in distinct clinical forms of leishmaniasis.

## Introduction

Experimental murine models have been used to elucidate mechanisms related to host immunity that regulate disease development. CBA mice develop a controlled disease in response to *L. major* infection, while this same mouse strain, in response to *L. amazonensis*, evolves to a severe form of disease with parasite dissemination (1). Macrophages are crucially important in the response to *Leishmania* infection, since these cells are not only considered as the primary host cell for *Leishmania* parasites, but also secrete cytokines in the assembling of an inflammatory response and release chemokines to recruit other immune cells to the site of infection or inflammation. CBA mouse MØ are capable of containing *L. major* parasite growth yet are susceptible to *L. amazonensis* (2). The CBA murine model of experimental leishmaniasis has shed light on several aspects of *Leishmania*–host interaction, as well as facilitated the identification of important immunoinflammatory molecules differentially produced in response to various parasite species despite their static genetic background.

Proteomics is an advanced large-scale technique used to identify and characterize global protein expression, allowing for the targeting of molecules in an infected sample in comparison to an uninfected reference control, both in intracellular microbes and host cells. This advanced technology has proven to be highly efficient in the study of individual molecules, as it permits the identification of hundreds or even thousands of signature proteins that characterize elements of microbial and host response during infection (1, 3–5). In the context of this large-scale analysis, bioinformatics contributes through the use of advanced computational algorithms to search databases [revised by Veras et al. (6)]. Several studies have demonstrated the power of proteomics with respect to providing valuable information regarding the identification of proteins and pathways involved in host-parasite interactions, both in parasites (7–10) as well as in the host (11, 12). More importantly, convincing evidence has been presented regarding the involvement of these identified molecules and pathways in the pathogenesis of leishmaniasis. In addition, some proteins have demonstrated potential as possible biomarkers for future prophylactic and therapeutic interventions (11, 13).

In intracellular microbial infections, the host oxidative stress response orchestrates the production of cytoprotective molecules, which can influence infection outcome (14). This cytoprotective process induces the activation of the cellular nuclear factor erythroid 2-related factor 2 (NRF2) encoded by the nfe2l2 gene, a transcription factor responsible for the regulation of host cellular redox balance (15, 16). Activated NRF2 binds to the consensus sequence of the antioxidant response element (ARE), which results in the transcription of a variety of genes, including glutamate-cysteine ligase (GCL), thioredoxin reductase 1 (Txnrd1), and NAD(P)H-qui-None oxidoreductase 1 (NQO1). This cytoprotective system is activated by the transcription of NRF2-mediated genes involved in iron metabolism, such as heme oxygenase-1 (HO-1) and the iron storage protein ferritin (17). HO-1, an essential enzyme involved in heme catabolism, as well as in the suppression of inflammatory responses, is induced in response to cellular stress and tissue damage. Oxidative stress activates the transcription factor NRF2, which binds to ARE, inducing the expression of protective antioxidant genes, including HO-1 (18–21). Previously, the major parasite surface glycoconjugate, lipophosphoglycan (LPG) was incriminated in HO-1 induction by parasite species that cause visceral leishmaniasis, *Leishmania infantum* (19). Recently, the ARE/NRF2 signaling pathway was found to be significantly upregulated in patients with cutaneous leishmaniasis (22). Since, some of those mechanisms are still unknown, use of the dichotomic model of CBA BMMØs infected by *L. amazonensis* and *L. major* (2) seems an interesting tool to clarify these issues.

Since iron is central to several metabolic processes for both prokaryotic and eukaryotic cells, iron homeostasis plays a major role in host–pathogen interaction (23–26). Accordingly, the ability of iron to transfer electrons necessary for metabolic processes results in the formation of highly reactive radicals by catalysis (27, 28). These radicals can act as signaling molecules in host cells, but can alternatively cause microbial intoxication, thereby damaging surrounding cells and tissues. In response to microbial infection, iron can act directly by imparting synergism toward the formation of anti-microbial radicals (27–30), or indirectly by modulating immune cell anti-microbial effector pathways (24). The latter type of response can result in reduced iron availability for microbes, and is dependent on the host cell response to cytokines that regulate the control of pathogen growth and establish an immune effector response (31).

Susceptibility to *L. amazonensis* has been demonstrated in CBA mice, in opposition to what was seen under *L. major* infection *in vivo* (1). In addition, CBA mouse MØ, which were found to be permissive to *L. amazonensis in vitro*, were observed to mitigate *L. major* infection (2). The present report employed proteomic analysis to determine the global macrophage response to *Leishmania* infection by comparing the differentially expressed proteins in CBA BMMØ infected or not with *L. amazonensis* or *L. major*. We hypothesized that protein signatures for BMMØ infected with either of these *Leishmania* strains would provide evidence regarding the proteins involved in infection development or control. Also, some functional experiments regarding iron metabolism were performed.

## Methods

### Ethics Statement

The CBA mice used in the present study were provided by the animal care facility at the Gonçalo Moniz Institute – Fiocruz - Bahia, following approval by the Institutional Animal Experimentation Review Board (CEUA) under protocol number 005/2014. Animals were kept and handled in accordance with the norms recommended by the International Guiding Principles for Biomedical Research Involving Animals; all experimental protocols complied with these guidelines, as well as all resolutions established by the Brazilian National Council for the Control of Animal Experimentation (CONCEA).

All protocols, analytic methods and material used in the present study are available upon request to all interested researchers.

### BMMØ Differentiation and Culturing

Mouse bone marrow macrophage precursors were harvested from CBA mice, differentiated into BMMØ, seeded onto 24-well plates (1 ml of 5 ×10^6^/ml suspension) 24 h prior to experimentation, and incubated in macrophage differentiation media (RPMI 20% FBS, 30% L cell-conditioned supernatant, 25 mM HEPES, 2 g/L sodium bicarbonate, 200 mM glutamine, and 1% ciprofloxacin) at 37°C/5% CO_2_. In brief, bone marrow from mouse femurs and tibias were flushed into RPMI 20% FBS media, cells were centrifuged and then seeded in Petri dishes overnight. The supernatant of these cells was then centrifuged at 300 × g for 10 min at 4°C, resuspended in media containing 30% L cell-conditioned supernatant and transferred to new plates for 7 days. For harvesting, cultures were incubated with 5 mM EDTA/PBS for 5 min at RT and then maintained overnight under 5% CO_2_ at 37°C in complete medium. All cell cultures were then washed and reincubated until the time of further analysis.

### *Leishmania* Culturing

*L. major* (strain MHOM/RI/-/WR-173) and *L. amazonensis* (strain MHOM/Br88/Ba-125) parasites were isolated from the lymph nodes of infected C57BL/6 mice. Axenic cultures of *L. amazonensis* or *L. major* promastigotes were maintained in culturing for up to six successive passages in Schneider's Insect Medium (Sigma, St. Louis, MO, United States) supplemented with 50 μg/mL gentamicin (Gibco, Grand Island, NY, United States) and 10% fetal bovine serum (Gibco, Grand Island, NY, United States), following a slightly modified previously described protocol (2). Axenic promastigotes were cultivated in an incubator at 24°C and monitored daily.

### Kinetics of BMMØ Infection With *L. amazonensis* or *L. major* Promastigotes

CBA mouse BMMØ were harvested and distributed at 5 ×10^6^ cells per well on 24-well plates, and then infected with stationary-phase *L. amazonensis* or *L. major* promastigotes at a parasite to macrophage ratio of 10:1. After 1.5, 3, or 6 h, cells were washed and fixed in 4% paraformaldehyde for 15 min. A separate group of cells were then washed to remove non-internalized parasites (after 6 h of infection) and these cells were reincubated for an additional 12 or 24 h at 37°C and then fixed. All cells were then subsequently mounted on coverslips using a ProLong Gold Antifade kit with DAPI (Life Technologies). The percentage of infected cells and the number of parasites per infected macrophage were determined by counting no <400 cells in random fields under an Olympus BX 51 fluorescence microscope using an 100x/1.4 objective in accordance with a protocol previously described by Huynh et al. (32).

### Preparation of Protein Extracts of BMMØ Infected With *L. amazonensis* or *L. major* Promastigotes and Processing for Tandem Liquid Chromatography-Mass Spectrometry

CBA mouse BMMØ were cultivated harvested and cultivated in DMEM complete medium (DMEM medium supplemented with 10% inactivated FCS, 2 g/L of sodium bicarbonate, 25 mM HEPES, 1 mM of glutamine, and 0.2% of ciprofloxacin) at 37°C in 5% CO_2_ for 18–24 h before infection at 5 ×10^6^ cells per well on six-well plates. Uninfected MØ were used as controls. In parallel, CBA MØ were infected with *L. major* or *L. amazonensis* stationary-phase promastigotes, at a parasite to macrophage ratio of 10:1. After a 6 h infection period, the control and infected cells were washed, and part of the cells was reincubated for an additional 24 h at 37°C. Finally, proteins were extracted from uninfected and infected MØ. Each experimental group was cultured in duplicate, and each biological experiment was repeated at least five times. All cell groups were, then, harvested and protein extraction was performed in 150 μL of lysis buffer (7 M urea, 2 M thiourea, 40 mM Tris, and 4% CHAPS), supplemented with a Protease Inhibitor Cocktail Tablets, Complete, Mini, EDTA-free Protease Inhibitor cocktail (Roche, IN, USA). The solutions were homogenized for 30 min and centrifuged at 13,800 × g for 20 min. After determining protein concentrations using a Bio-Rad Protein Assay Kit (Bio-Rad Laboratories Inc., Hercules, CA, USA), the contaminants were removed using a 2D clean-up kit (GE Healthcare, Waukesha, WI, USA). Following purification, the samples were resuspended in a solution of 8 M urea and 5 mM dithiothreitol (DTT) and incubated for 1–2 h. Subsequently, a solution containing 200 mM iodoacetamide (IAA) in 1 M ammonium bicarbonate (NH_4_HCO_3_) was mixed into the sample solution and incubated for 45 min at room temperature under total darkness. Next, the samples were incubated with 200 mM of DTT for 20 min at room temperature. All samples were then diluted in 50 mM of NH_4_HCO_3_ and 2 mM of CaCl_2_, followed by the addition of a trypsin solution for protein digestion and the generation of tryptic peptides at a ratio of 1:30 (trypsin: protein). Digestion occurred over 16–18 h at 37°C and was interrupted by the addition of a 10% trifluoroacetic acid solution and the subsequent pH adjustment to 5.0. The resulting tryptic peptides were desalted on C8 cartridges (Michrom BioResources, Auburn, CA, USA) and subjected to 2D Nano tandem liquid chromatography-mass spectrometry (LC-MS/MS) analysis using a Dionex nano-LC system (Dionex Corporation, Sunnyvale, CA, USA). To perform first-dimension separation, a 300 μm ID SCX column (PolyLC Polysulfoethyl A, 150 ×0.3 mm, 5 μm, 200 A) was used with a 15-step gradient (0–100%, pH 3.6–6.5) of ammonium formate (generated in-house, 0.8 M solution), with each step lasting 1 h, at a flow rate of 5 μL/min. The peptides eluted from the SCX column were trapped on a C4 precolumn (Dionex PepMap300, 5 μm, 300 A, 300 μm ID ×5 mm), desalted [0.1% formic acid, 2% acetonitrile (ACN)] and separated on a 75 μm ID C18 column (Dionex NAN75-15-15-03-C18 PepMap100 stationary phase, 3 μm) using an acetonitrile gradient at a flow rate of 200 nL/min. Finally, the eluted peptides were electrosprayed, using a potential of 1.8–2.2 kV, onto an LCQ Deca XP(Plus) ion trap mass spectrometer (Thermo Finnigan Corporation, San Jose, CA, USA) in data-dependent mode. A full-scan MS spectra survey was acquired at m/z from 350 to 2,000, after which the four most abundant ions were selected and fragmented for the production of tandem mass spectra. Any target ions previously selected twice for MS/MS were dynamically excluded for 3 min. Peptide dissociation was performed at 35% of the normalized collision energy, and the MS/MS spectra were recorded in profile mode.

### Protein Identification

In order to differentiate between macrophage and *Leishmania* proteins, the MS/MS spectra were searched against both the *Mus musculus* and *L. amazonensis* local protein databases using the SEQUEST algorithm in Bioworks v3.2 software. The X_corr_ and ΔCn threshold values for a 1% false discovery rate (FDR) were used to obtain the peptide ID list. The FDR was calculated using a reverse database and the modification parameters were set to +57.02146 for cysteine alkylation and +15.99492 for methionine oxidation. The spectra search allowed for a maximum mass deviation of 3 amu and two missed cleavage sites. The only peptides selected for further analysis were those identified as possessing fully tryptic termini with cross-correlation scores >1.9 for single-charged, 2.3 for double-charged, and 3.75 for triple-charged, with a delta-correlation score over 0.1 and a probability score lower than 1 ×10^−5^. All peptides that met these criteria were included in the differential expression analysis, in which a quantitative metric was applied. For data normalization, the counts corresponding to each peptide were divided by the total sum of all the peptide counts in a given sample, and then divided by the highest total sum of all the peptide counts among all samples assessed throughout the entire experiment. These results were then transformed to log2 and two-way ANOVA analysis was applied, in which Log2 fold change (Log_2_FC) and FDR (*q*-value) were assessed.

### Datamining Analysis

Using Weka software, the CfsSubsetEval and BestFirst features were used to select attributes in the dataset strongly associated with experiment conditions (Weka 3.9.3, University of Waikato). Principal Component Analysis (PCA) was employed to reduce dimensionality of selected attributes in the dataset strongly associated with experiment conditions (Weka 3.9.3, University of Waikato). Using Orange software, the Find Informative Projections feature was used to identify well-separated patterns/clusters within the dataset based on the new attributes generated by PCA (Orange 3.17.0, University of Ljubljana). IPA software was used for enrichment analysis to identify relevant biological pathways (QIAGEN Inc., https://www.qiagenbioinformatics.com/products/ingenuity-pathway-analysis).

### Expression of NRF2, p62, HO-1, and Ferritin in BMMØ Infected With *L. amazonensis, L. major* Promastigotes or Stimulated With LPG

For these experiments, Western blotting was used to calculate the ratios between the amounts of NRF2, p62, HO-1, ferritin, and actin in the extracts of CBA BMMØ. MØ were infected with *L. amazonensis* or *L. major*, or stimulated with purified intact LPG extract at 10 μg/mL from each of these *Leishmania* species (for HO-1 experiments) for 6 h, and a separate group of cells were then washed to remove non-internalized parasites and reincubated for an additional 24 or 48 h. LPG extraction and purification was performed in accordance with a protocol previously described by Nogueira et al. (33). Cobalt protophorphyrin IX (CoPP), a pharmacologic inductor of HO-1, was used as positive control of HO-1 expression. For total protein extraction (p62, HO-1, ferritin and actin), BMMØ (2 ×10^6^ cells) were washed twice with ice-cold PBS and then lysed in 100 μl of lysis buffer (50 mM Tris-HCl, pH 7.5, 5 mM EDTA, 10 mM EGTA, 50 mM NaF, 20 mM β-glycerophosphate, 250 mM NaCl, 0.1% Triton X-100, 1 μg/ml BSA, and a 1:100 dilution of protease inhibitor cocktail, Sigma, St. Louis, MO, USA). For nuclear protein extraction (NRF2), cells were washed twice with 1x PBS and then lysed with 100 μL of buffer A (HEPES 10 mM pH 7.9. 10 mM KCl, 0.1 mM EDTA, 0.1 mM EGTA, NP-40 0, 25% (v/v); cocktail of protease inhibitors) for 10 min on ice. All lysed cells were then centrifuged at 14,000 g for 1 min at 4°C, and the pellet was resuspended in 60 μL of buffer C (20 mM HEPES pH 7.9, 0.4 M NaCl, 1 mM EDTA, 1 mM EGTA, 20% glycerol, protease inhibitor cocktail) and incubated on ice for 20 min. The lysate was centrifuged at 14,000 g for 5 min, and the supernatant containing nuclear proteins was collected in a new tube. Protein extract was subjected to electrophoresis on 10% SDS-polyacrylamide gel and transferred to nitrocellulose membranes (Amersham Biosciences, Piscataway, NJ, USA). After blocking with 5% non-fat dry milk in TBS with 0.1% Tween-20 (TBS-T), blots were incubated overnight with antibodies against NRF2, p62, HO-1, ferritin, and β-actin, followed by secondary antibody labeling. All membranes were then washed thrice with 0.1% TBS-T and blots were developed with an ECL Chemiluminescence Kit (Thermo Fisher Scientific, Rockford, IL, United States). Bands were detected using a Luminescent Image Analyzer and Image Quant Las 4000 software. Densitometry quantification was performed using Image J software. In addition, HO-1 validation was also performed by ELISA in accordance with a protocol previously described (19).

### Quantification of Nitric Oxide (NO) Production

To assess the differential production of NO by *L. amazonensis-* or *L. major*- infected BMMØ, cells were primed with 50 UI/mL IFN-γ (R&D Systems, Minneapolis, MN, USA) for 24 h and subsequently infected. After 6 h part of the cells was fixed and part was washed and reincubated in DMEM medium containing IFN-γ for an additional 24 h. Infected cells incubated in IFN-γ-free medium were used as a negative control. NO production was measured in culture supernatants by determining nitrite accumulation using the Griess reaction.

### Binding and Internalization of HoloTf by Infected BMMØ

For holoTf uptake assays, BMMØ were plated on 24-well plates at a concentration of 2 ×10^5^ per well, infected with *L. amazonensis or L. major* (10:1) or left uninfected. After 6 h part of the cells was fixed and part was washed and then reincubated with 300 nM of holoTf-Texas Red (Molecular Probes) at 4°C for 30 min in complete DMEM medium, in which FCS was replaced with 1% de Nutridoma-SP (Roche). Cells were then washed with cold saline and reincubated in holoTf-free medium for an additional 40 min at 37°C. Finally, cells were washed again with cold saline and either fixed with 4% paraformaldehyde for 15 min or reincubated for an additional 24 h before fixation. Coverslips were mounted using a ProLong Gold Antifade kit containing DAPI (Life Technologies). Images were acquired on a Leica SP8 confocal microscope and processed using ImageJ software. Fluorescence intensity from 30 cells from each group after binding or internalization of holoTf was quantified in whole cell and parasitophorous vacuoles of each infected macrophage using ImageJ software. Statistical differences were evaluated using the Student's *t-*test (^*^p < 0.05). Data are representative of three independent experiments.

### Statistical Analysis

All statistical analysis was carried out using GraphPad Prism Software (version Mac 5.0c). After evaluating the normality of data, the unpaired Student's *t-test* or the Mann-Whitney test were used for comparisons between two groups, for Gaussian and non-Gaussian distributions. For comparisons among three or more groups, one-way ANOVA was used for Gaussian distribution, while for non-Gaussian distribution, the Kruskal-Wallis non-parametric test was employed. Differences among tested groups were considered statistically significant when *p* < 0.05.

## Results

### Kinetic Model of BMMØ Infection With *L. amazonensis* or *L. major* Promastigotes

**Figure 1:**
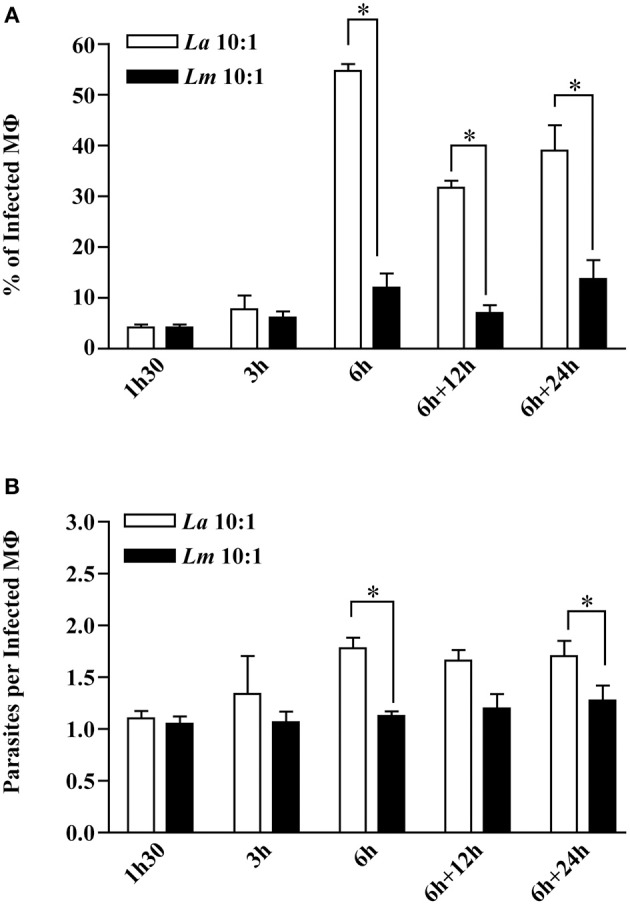
Kinetics of CBA BMMØ infection by *L. amazonensis* or *L. major*. BMMØ were infected as described under Materials and Methods. Cells were fixed and then DAPI-stained after 1.5, 3, or 6 h. Additionally, other cells infected for 6 h were washed and re-incubated for an additional 12 or 24 h. **(A)** Percentage of infected BMMØ and **(B)** amastigotes per macrophage were quantified by fluorescent microscopy. Statistical differences were evaluated using the Mann-Whitney test (*p < 0.05). Data represent means ± SE of three independent experiments.

Kinetic analysis of BMMØ infection involving *L. amazonensis* or *L. major* promastigotes revealed that the percentage of infected MØ and the number of parasites per infected cell were similar in both infected cell groups at 1.5 and 3 h after parasites were added to cell cultures (Mann-Whitney, *p* < 0.05, Figures 1A,B). At 6 h of infection, the percentages of infected BMMØ for *L. amazonensis* and *L. major* infection were different, respectively, 54.88 and 12.00%. After reincubation times, the percentage *L. amazonensis*- and *L. major*-infected MØ maintained these differences, respectively, 31.69 vs. 7.00% at 12 h, and 38.94 and 13.69% at 24 h (Mann-Whitney, *p* < 0.05, Figure 1A). Similarly, the average number of parasites per *L. amazonensis*- or *L. major*-infected macrophage were distinct, respectively: 1.78 vs. 1.13 at 6 h; 1.70 vs. 1.27 at 24 h (Mann-Whitney, *p* < 0.05, Figure 1B).

### LC-MS/MS Analysis and Datamining Identified Differentially Expressed Proteins Associated With *Leishmania* Infection in Murine MØ

**Figure 2:**
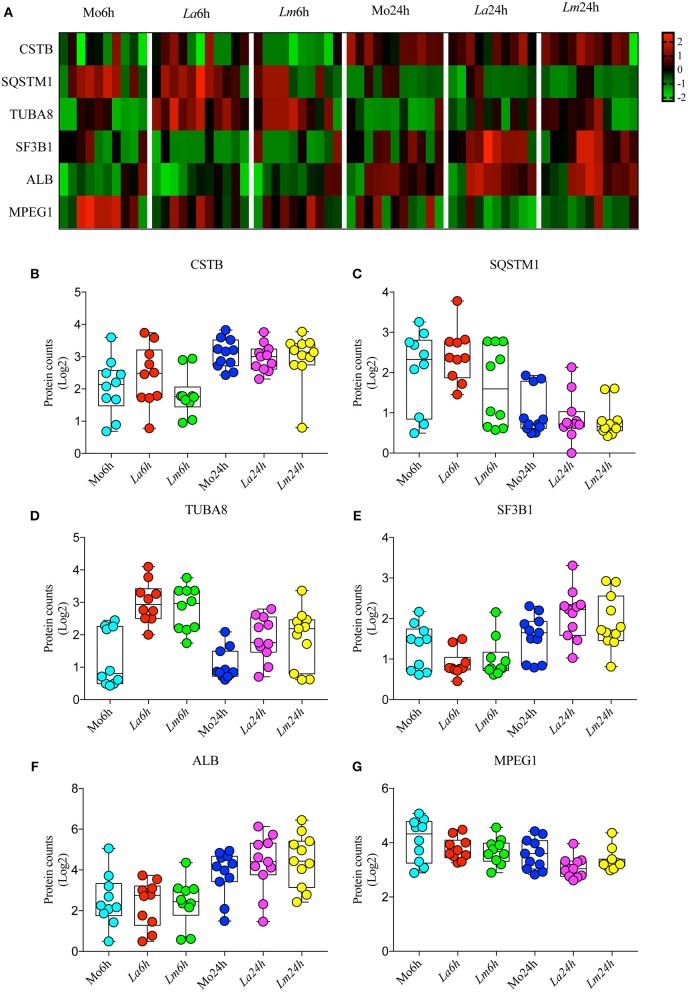
Selected proteins strongly associated with experimental conditions. **(A)** Heatmap showing representative Z-scores based on selected protein counts corresponding to each experimental condition replicate. **(B–G)** Quantitative values of log2 protein counts from each experimental condition represented by box plots and dot plots.

**Figure 3:**
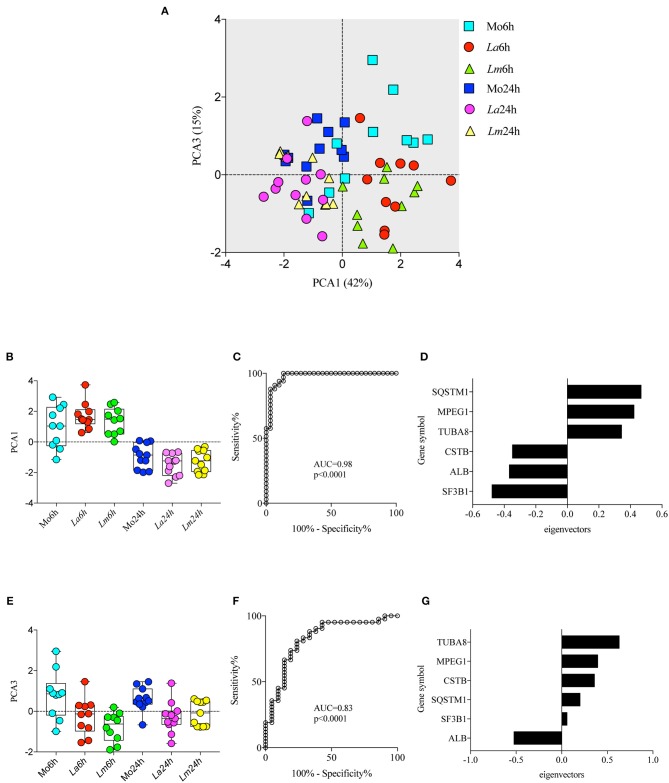
Principal Component Analysis (PCA) associated with experimental conditions. PCA was employed to reduce dimensionality and extract principal components using linear combinations of the six selected proteins in the dataset **(A)** Principal component 1 (PCA1) and principal component 3 (PCA3) scores are depicted in a two-dimensional scatterplot for each sample. Associations between cell culturing times and PCA1 scores **(B,C)** and *Leishmania* infection conditions and PCA3 scores **(E,F)** are represented by box plot and dot plot graphs **(B,E)** and ROC curve analysis **(C,F)**. **(D,G)** Bar graph depicting protein eigenvectors values for PCA1 **(D)** and PCA3 **(G)**. PCA1 scores are characterized by the positive eigenvector values of SQSTM1, MPEG1, and TUBA8, and the negative eigenvector values of CSTB, ALB, and SF3B1. PCA3 scores are characterized by the positive eigenvector values of TUBA8, MPEG1, and CSTB, and the negative eigenvector values of SQSTM1, SF3B1, and ALB.

To investigate the differences in BMMØ susceptibility between *L. amazonensis* and *L. major* infection, a proteomic approach was employed to obtain global protein cell signatures at an initial time point (6 h) and at a later time point (24 h) following parasite washout. BMMØ protein identification and characterization from ten independent experiments were performed using LC-MS/MS with a MudPIT approach. The SEQUEST algorithm revealed a total of 2,838 significantly expressed proteins. Subsequently, data pre-processing was performed to normalize the samples before determining the protein signatures for CBA BMMØ infected with each *Leishmania* species. Using a supervised learning method (CfsSubsetEval and BestFirst) for filtering proteins, six proteins [cystatin B (CSTB), sequestosome 1 (SQSTM1 or p62), tubulin alpha 8 (TUBA8), splicing factor 3b subunit 1 (SF3B1), albumin (ALB), and macrophage expressed 1 (MPEG1)] were identified as strongly associated with experimental conditions (Figure 2). To evaluate the global significance of these proteins in the context of *Leishmania* infection, an unsupervised method (Principal Component Analysis) reduced the dimensionality of the selected proteins and generated new attributes, including PCA1 and PCA3. Using the Find Informative Projection option, PCA1 (42%) and PCA3 (15%) combined were able to account for 57% of the variance of the entire dataset (Figure 3A). Of note, using the eigenvector values associated with PCA1 and PCA3, we were able to confidently classify the following conditions with each feature: PCA1, cell culturing times (6 ×24 h) (ROC curve analysis, AUC = 0.98, *p* < 0.0001) (Figures 3B–D); PCA3, *Leishmania* infection conditions (uninfected MØ × *L. amazonensis-* or *L. major*-infected MØ) (ROC curve analysis, AUC = 0.83, *p* < 0.0001) (Figures 3E–G).

### Biological Enrichment Analyses in Differentially Expressed Proteins Associated With *Leishmania* Infection in Murine MØ Reveal a NRF2-dependent Mechanism

**Figure 4:**
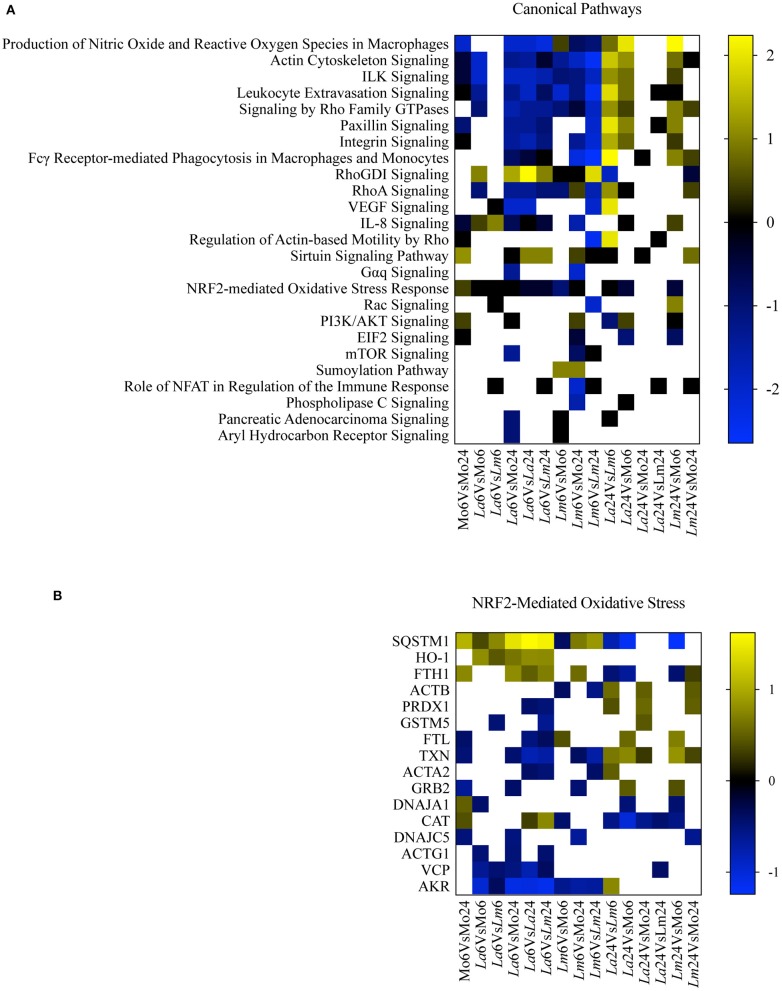
Canonical pathway enrichment analysis. **(A)** Heatmap representing enriched canonical pathways produced by IPA from 15 datasets generated from an all-vs.-all comparison of the study conditions: Z score = −1 to 1 and |–Log10(*p*-value)| > 1.3. Blue blocks and yellow blocks represent inhibited and activated canonical pathways, respectively. White blocks represent canonical pathways without significance in a given experimental comparison. **(B)** Heatmap representing Log2 fold changes of significantly regulated genes (multiple comparison two-way ANOVA, *q* < 0.05 and |Log_2_FC| > 0.2) present in the dataset included in the NRF2-Mediated Oxidative Stress canonical pathway. Blue blocks and yellow blocks represent down-regulated and up-regulated genes, respectively. White blocks are representative of genes not significantly regulated in a given experimental comparison.

**Figure 5:**
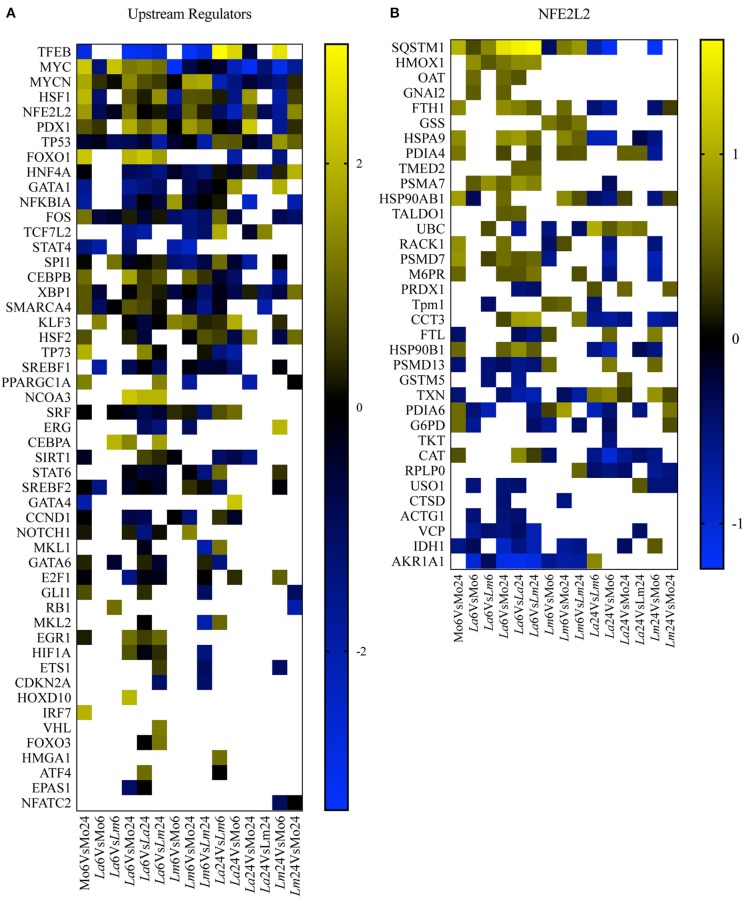
UTF enrichment analysis. **(A)** Heatmap representing enriched UTFs, filtered by transcription regulators with Z scores = −1 to 1 and |–Log10(*p*-value)| > 1.3, obtained by IPA using 15 datasets generated from an all-vs.-all comparison of the study conditions. Blue blocks and yellow blocks represent inhibited and activated UTFs, respectively. White blocks represent UTFs without significance in a given experimental comparison. **(B)** Heatmap representing Log2 fold changes in significantly regulated genes (multiple comparison two-way ANOVA, *q* < 0.05 and |Log_2_FC| > 0.2) present in the dataset of the NRF2/NFE2L2 UTF pathway. Blue blocks and yellow blocks represent down-regulated and up-regulated genes, respectively. White blocks are representative of genes not significantly regulated in a given experimental comparison.

To perform a biological interpretation of the six identified proteins IPA was used for enrichment analysis to identify CPs and UTFs filtered by Transcription regulators in three steps. Firstly, biological enrichment analysis using IPA identified 21 CPs and 142 possible UTFs containing at least one of the six genes (data not shown). Next, IPA was performed in 15 datasets generated from an all-vs.-all comparisons of the study conditions (total of 365 statistically significant mapped proteins, multiple comparison two-way ANOVA, *q* < 0.05 and |Log_2_FC| > 0.2, Supplementary Table 1) and identified 25 CPs and 51 possible UTFs using a Z score = −1 to 1 and |–Log10(*p*-value)| > 1.3 (Figures 4A, 5A). Finally, to filter the possibilities for validation, we overlaid the biological enrichment analysis results from the six proteins with the 15 datasets generated, which revealed that only 4 CPs (Production of Nitric Oxide and Reactive Oxygen Species in MØ, IL-8 Signaling, Sirtuin Signaling Pathway, and NRF2-mediated Oxidative Stress Response) and 7 UTFs [MYC proto-oncogene (MYC), transcription factor 7 like 2 (TCF7L2), promyelocytic leukemia (PML), CCAAT enhancer binding protein alpha (CEBPA), NFE2L2 (NRF2), tumor protein p53 (TP53) and signal transducer, and activator of transcription 6 (STAT6)] were strongly involved in the infection process. Among these findings, the NFE2L2 signature was the only molecule both present in both CPs and UTFs. The NRF2 CP and the NRF2 UTF analyses identified a group of molecules besides SQSMT1 (Figures 4B, 5B). The involvement of the iron metabolism pathway was demonstrated by the presence of HO-1 and ferritin, probably, recruited by NRF2. Of note, HO-1 was strongly associated only with *L. amazonensis* infection at 6 h (data not shown), while ferritin was regulated among both parasites (data not shown). These differently expressed proteins were then validated by ELISA or Western-blotting at time points varying from 6 to 48 h post-infection.

### NRF2 Expression by *L. amazonensis*- or *L. major*-Infected BMMØ

**Figure 6:**
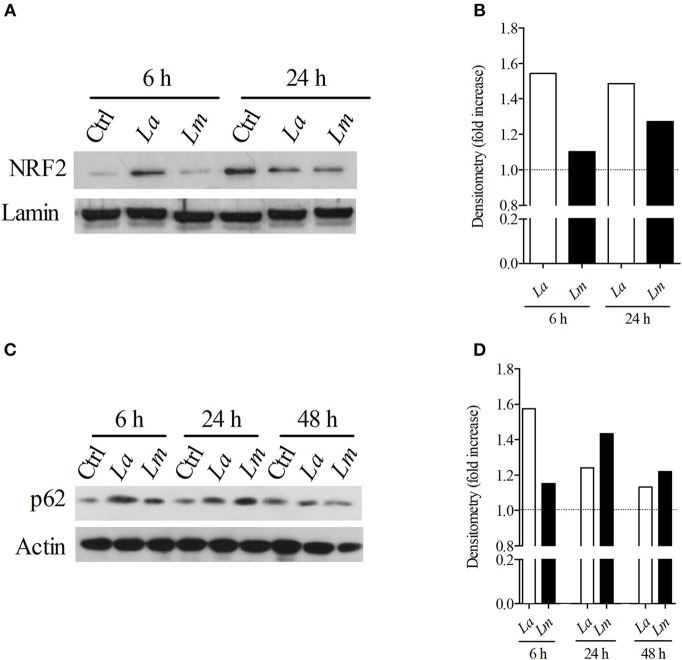
*L. amazonensis* infection induces higher levels of NRF2 and p62 in CBA BMMØ. NRF2 and p62 protein levels were evaluated following *L. amazonensis* and *L. major* infection. BMMØ were infected or not with *L. amazonensis* or *L. major* as described in Materials and Methods. After 6, 24, or 48 h of infection, MØ were washed to remove extracellular parasites and protein levels were quantified by Western blotting. **(A)** Effect of *L. amazonensis* or *L. major* infection on NRF2 protein levels. Anti-laminin antibodies were used as loading controls. **(B)** NRF2/laminin fold increase ratios in comparison to control levels (dashed line), as determined by densitometry. Results shown in **(A,B)** are representative of two independent experiments. **(C)** Effect of *L. amazonensis* or *L. major* infection on p62 protein levels. Anti-actin antibodies were used as loading controls. **(D)** p62/actin fold increase ratios in comparison to control levels (dashed line), as determined by densitometry. Results shown in **(C,D)** are representative of three independent experiments.

Recently, it was demonstrated that *L. amazonensis* and *L. braziliensis* induce NRF2 expression via the activation of the protein kinase R (PKR) pathway. Importantly, NRF2 activation resulted in infection tolerance *in vivo*, which enhanced intracellular pathogen survival (22). Accordingly, here we sought to investigate whether NRF2 would be differentially expressed by *L. amazonensis* and *L. major*-infected cells. Using Western-blotting, we detected elevated levels of NRF2 transcription factor translocated to the nuclei (Figures 6A,B) and p62 protein (Figures 6C,D) at 6 h in both *L. amazonensis*- and *L. major*-infected cells in comparison to uninfected control BMMØ, although to a greater extent in *L. amazonensis*-infected BMMØ, which is the strain highly virulent to CBA mouse MØ (Figure 6).

### Validation of Proteins Differentially Expressed by *L. amazonensis*- and *L. major*-Infected BMMØ

**Figure 7:**
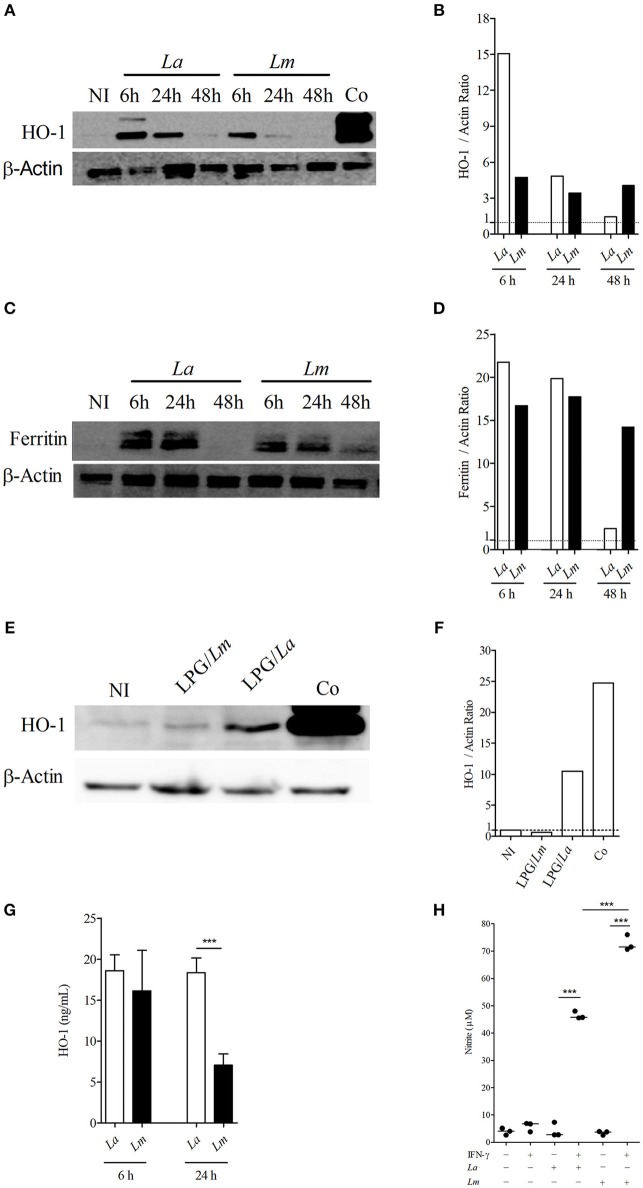
*Leishmania* spp. infection modulates levels of HO-1, ferritin and NO production in CBA BMMØ. Effect of *L. amazonensis* and *L. major* infection on HO-1 and ferritin protein levels, as well as NO production. BMMØ were infected or not with *L. amazonensis* or *L. major*, or incubated with purified LPG at 10 μg/mL from parasites from these species as described in Materials and Methods. **(A,C,E)** Cobalt protoporphyrin IX stimulation was used as positive control. Six or 24 h after infection, MØ were washed to remove extracellular parasites and protein levels were quantified by Western blotting. Actin was used as a loading control. **(B,D,F)** HO-1/actin and ferritin/actin fold increase ratios in comparison to control levels (dashed line), as determined by densitometry. Results shown in **(B,D,F)** are representative of three independent experiments. **(G)** Effect of *L. amazonensis* and *L. major* infection on HO-1 production by ELISA. Statistical differences were evaluated using the One-Way ANOVA Tukey's Multiple Comparison Test. ***p < 0.0001. Data are representative of means ± SE from five experiments. **(H)** Effect of *L. amazonensis* and *L. major* infection on BMMØ NO production. Macrophage NO production was measured by detecting nitrite in culture supernatants. Statistical differences were evaluated using the Student's *t-*test. ***p < 0.0001. Data are representative of means ± SE three independent experiments.

Western-blotting analysis confirmed that NFR2 activated different signaling pathways in BMMØ infected with each strain of *Leishmania*. IPA analysis indicated that NRF2 is an upstream regulator known to be involved in the activation of several proteins identified by SEQUEST analysis, of which HO-1 was validated herein. Accordingly, the protein involved in antioxidant response, HO-1, was found to be highly expressed at 6 h by *L. amazonensis*-infected BMMØ (Figures 7A,B), while increased levels of the main iron storage protein, ferritin, was detected at 48 h in *L. major*-infected MØ (Figures 7C,D). The Western-blotting assay showed higher expression of HO-1 in *L. amazonensis*-infected MØ, which was confirmed by an ELISA that specifically detected higher amounts of HO-1 in cell extracts at 24 h after infection (Figure 7G). Also, BMMØ incubated with LPG isolated from *L. amazonensis* expressed higher levels of HO-1, when compared to those incubated with LPG isolated from *L. major* parasites (Figures 7E,F).

### Nitric Oxide (NO) Production by BMMØ Infected With *L. amazonensis* or *L. major* Promastigotes

Since there is clear evidence linking the nitric oxide production by infected cells, HO-1 expression and the capability of host cells to control *Leishmania* infection *in vivo*, as well as *in vitro* (19), we decided to investigate whether the control of *L. major* induced by CBA BMMØ would be associated with the production of microbicidal molecules to a greater extent than in *L. amazonensis*-infected BMMØ. All control cells, whether uninfected, infected but not stimulated, or simply stimulated with IFN-γ (100 UI/mL) produced low levels of NO, without any significant differences. Only infected cells stimulated with IFN-γ produced increased amounts of NO, and 1.5 times more NO was detected in *L. major*-infected BMMØ than in *L. amazonensis*-infected cells at 24 h after infection (Figure 7H).

### Functional Evaluation of Proteins Involved in Iron Metabolism

Evidence has indicated a link between the modulation of proteins that participate in the metabolism of iron and susceptibility to *Leishmania* infection in several experimental models of leishmaniasis (34). Herein we found that two proteins involved in iron metabolism, HO-1 and ferritin, were differentially expressed by *L. amazonensis*- and *L. major*-infected BMMØ. In light of this, the CBA mouse model was employed to further investigate the role played by iron metabolism elements in the inflammatory response to *Leishmania* infection.

**Figure 8:**
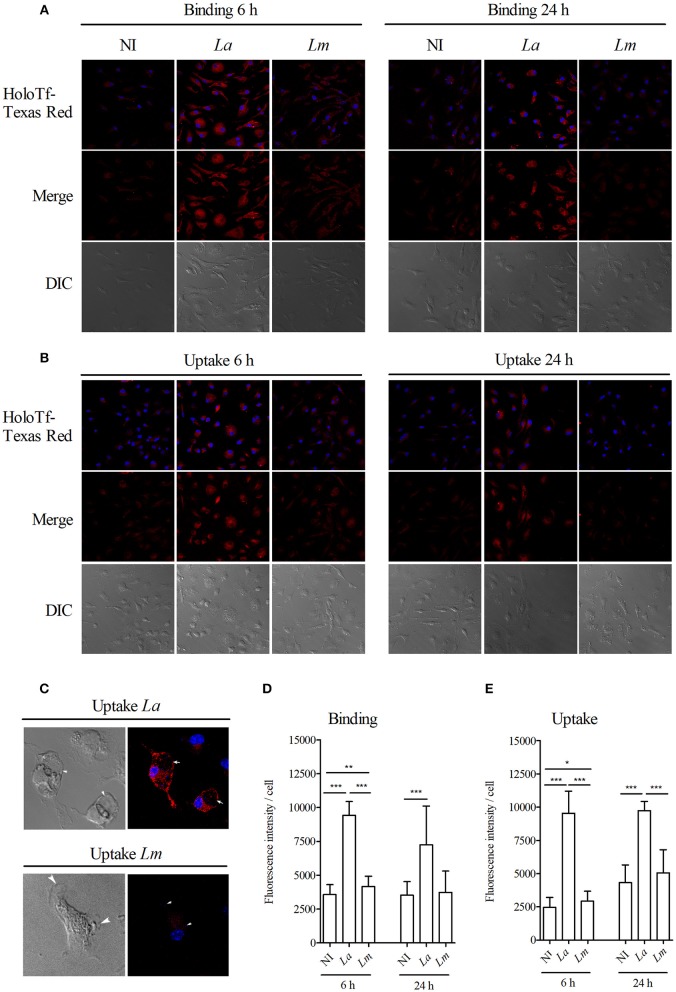
*L. amazonensis* induces higher holoTf uptake than *L. major* in BMMØ. BMMØ either uninfected, or infected with *L. amazonensis or L. major* for 6 or 24 h, were incubated with **(A)** holoTf-Texas Red for 30 min at 4°C to allow binding or **(B)** at 37°C to allow internalization, followed by reincubation for an additional 40 min in holoTf-free medium and then analyzed by confocal microscopy. Fluorescence intensity from 30 cells from each group after binding **(D)** or internalization **(E)** of holoTf was quantified using ImageJ software. **(C)** Images illustrating *L. amazonensis-* and *L. major*-induced parasitophorous vacuoles labeled with holoTf. Statistical differences were evaluated using the Student's *t-*test (*p < 0.05, **p < 0.001, ***p < 0.0001). Data are representative of three independent experiments.

Next, we attempted to assess whether other proteins than HO-1 and ferritin, which are also involved in iron metabolism, are differentially influencing *L. amazonensis*- and *L. major* infection of CBA BMMΦ. Using confocal microscopy, we found increased binding (Figures 8A,C) and greater uptake (Figures 8B,D) of fluorescent holoTf in *L. amazonensis*-infected BMMΦ. More interestingly, fluorescent-labeled holoTf was also detected at higher levels in the large parasitophorous vacuoles (Figure 8E) induced by *L. amazonensis*, in comparison to those found in the smaller vacuoles induced by *L. major*, for up to 24 h after infection. In sum, these findings indicate holoTf-mediated endocytosis was higher in the *L. amazonensis*-infected cells.

## Discussion

Advances in proteomics have greatly contributed to the identification of biological pathways, both in the parasite and host, allowing for a more global and integrated view of biological processes (4). In the field of *Leishmania* research, many proteomic studies have focused on a variety of aspects related to parasite biology and host interactions, such as parasite life-cycle, drug resistance mechanisms, identification of immunogenic proteins for vaccine development and the identification of targets for chemotherapeutic treatment, as well as biomarkers that hold potential for use in diagnosis (9–11, 35–39). However, the mechanisms involved in interactions between *Leishmania* parasites and the host, as well as the factors that drive disease outcome, warrant comprehensive investigation [revised by Veras et al. (6)].

Using a proteomic approach, the present study demonstrated that *L. amazonensis* and *L. major* modulate the expression of proteins differently in CBA MØ. A total of 2,838 proteins were initially identified in BMMØ using the SEQUEST algorithm. Data mining analyses and validation assays were then performed, and IPA was employed to perform biological enrichment analysis using the 15 datasets generated from all-vs.-all comparisons of the experimental conditions. IPA revealed that NRF2 transcriptional factor signatures, present in one of the 4 CPs and one of the 7 UTFs, were strongly associated with *Leishmania* infection. In consonance, a recent study described that *Leishmania* induces NRF2 expression in MØ via protein kinase R *in vitro*, as well as in tissues of human cutaneous leishmaniasis. In addition, transcriptomic analysis highlighted the importance of NRF2 signaling in cutaneous leishmaniasis (22).

The Great Oxygenation Event (GOE) resulted in increased levels of O_2_, which oxidized ferrous iron (Fe^2+^), thereby resulting in the rusting of the earth. The development of antistress systems was critical for reducing exposure to these toxic elements during early evolutionary development. Among these critical systems, the Kelch-like ECH-associated protein 1 (KEAP1)-NRF2 system emerges as one mainly responsible for antioxidant defenses, with NRF2 being the master transcription factor that possibly appeared around the time of the GOE (40). KEAP1-NRF2 is a delicate detoxification system in mammalian cells that exerts both cytoprotective and antioxidant functions, as well as deleterious effects by producing free radicals that can be regulated in several steps involved in iron metabolism (15–17, 41).

Cumulative evidence indicates that iron metabolism influences parasite survival within parasitophorous vacuoles (42, 43). *Leishmania* parasite-host cell interaction triggers a finely regulated response that can result in either a beneficial effect, iron acquisition and usage by *Leishmania*, which is essential to parasite multiplication, or can generate byproducts that accommodate reactive free radicals, which are toxic to intracellular parasites (44, 45). NO^•^ is a known intracellular secondary messenger that can also react with superoxide anion (O2^**·**−^) to form peroxynitrite (ONOO-) by way of an enzyme-independent mechanism. ONOO- is a highly reactive molecule that interacts with most biologic molecules, including Fe-S clusters, often causing reversible/irreversible cell damage (45). Previously, we also demonstrated that *L. amazonensis*-infected MØ induced lower amounts of ROS in comparison with *L. major* (46). Herein, we showed that *L. amazonensis*-infected MØ produced lower levels of NO in comparison with *L. major*. Consistently with our results, NRF2-deficient MØ presented increased levels of ROS/RNS and reduced expression of Sod1, a NRF2-dependent gene, which resulted in reduced parasite load (22). Although these results reinforce the notion that NRF2 activation and translocation to the nucleus is associated with a reduced oxidative response in *L. amazonensis* infection, the mechanisms and metabolic processes involved in the growth and killing of the parasite are not similar to those involved in *L. major*. Thus, it is possible that factors other than oxidative response play an important role in *Leishmania* spp infection outcome. In the case of *L. amazonensis*, high expression of the tryparedoxin peroxidase isoform contributes to parasite ability to adapt to and antagonize the hostile microenvironment of macrophage-induced parasitophorous vacuoles, providing an alternative explanation for persistent infection in the mammalian host (47).

Host MØ can regulate the production of deleterious iron byproducts generated from several steps of iron metabolism, together with the participation of some proteins, we found in the present study to be highly expressed by *L. amazonensis*-infected BMMØ. Studies have consistently shown that enhanced HO-1 expression is associated with increased parasite load, as well as the activation of an anti-inflammatory response in conjunction with reduced levels of inflammatory cytokines, such as TNF-α and IL-12. These effects are orchestrated by Nrf2-promoter activation, which also results in increased levels of Cu/Zn superoxide dismutase expression in human monocytes (19, 22, 44). Previous study has shown that either parasites and its LPG induced HO-1 in MØ infected with the viscerotropic species *L. infantum* (19). Herein, we observed that higher levels of HO-1 was induced by *L. amazonensis* and its LPG compared to *L. major*. In accordance with these observations, it is possible that this different responses may be a result of LPG polymorphisms in those species (48–50). Further evidence presented herein links the involvement of the iron metabolism pathway with elevated HO-1 in *L. amazonensis*-infected cells. In addition, BMMØ incubated with *L. amazonensis* LPG were also shown to induce higher levels of HO-1 expression. Furthermore, the *L. amazonensis*-induced uptake of HoloTf in BMMØ was higher than that induced by *L. major*, and HoloTf was also detected at higher levels in vacuoles induced by *L. amazonensis*. These findings corroborate a previous study that showed holoTf-labeled endosomes preferentially fuse with *L. amazonensis*-induced parasitophorous vacuoles (51).

Altogether, in contrast to *L. major*-infected BMMØ, *L. amazonensis*-infected BMMØ exhibited increased expression of NRF2 and anti-oxidant HO-1, together with higher levels of holoTf in parasitophorous vacuoles, which may contribute to the persistence of *Leishmania amazonensis* infection. In this context, in addition to parasite factors, different protein signatures are triggered in CBA BMMØ in response to *L. amazonensis* and *L. major* infection, which could play a role in the outcome of distinct clinical forms of leishmaniasis.

## Data Availability

The SEQUEST output file can be found in the Dryad Digital Repository: https://doi.org/10.5061/dryad.tr4185n.

## Ethics Statement

The CBA mice used in the present study were provided by the animal care facility at the Gonçalo Moniz Institute–Fiocruz-Bahia, following approval by the Institutional Animal Experimentation Review Board (CEUA) under protocol number 005/2014. Animals were kept and handled in accordance with the norms recommended by the International Guiding Principles for Biomedical Research Involving Animals; all experimental protocols complied with these guidelines, as well as all resolutions established by the Brazilian National Council for the Control of Animal Experimentation (CONCEA).

All protocols, analytic methods and material used in the present study are available upon request to all interested researchers.

## Author Contributions

JdM and PV conceived and designed the experiments. JdM, RK, CO, AP, TdA, FRLM, AR, and AL performed and analyzed the proteomic experiments. NL, JL, ÁV, RS, UL, and VB performed and analyzed the validation experiments. JdM, RK, RS, GB, MK, FKM, UL, JR, VB, and PV contributed with reagents, materials, and analysis tools. JdM and PV wrote the manuscript. All authors contributed to manuscript elaboration and revision and approved the final version prior to submission.

### Conflict of Interest Statement

The authors declare that the research was conducted in the absence of any commercial or financial relationships that could be construed as a potential conflict of interest.
